# Analysis and performance assessment of a fragment retransmission scheme for energy efficient IEEE 802.11 WLANs

**DOI:** 10.1186/s40064-016-3023-6

**Published:** 2016-08-24

**Authors:** Prosper Mafole, Masayoshi Aritsugi

**Affiliations:** 1 Department of Electronics and Telecommunication Engineering, College of Information and Communication Technologies, University of Dar es Salaam, Dar es Salaam, Tanzania; 2 Big Data Science and Technology, Division of Environmental Science, Faculty of Advanced Science and Technology, Kumamoto University, Kumamoto, Japan

**Keywords:** CSMA, IEEE 802.11 fragmentation, Energy efficiency, Mathematical analysis, Simulation

## Abstract

Backoff-free fragment retransmission (BFFR) scheme enhances the performance of legacy MAC layer fragmentation by eliminating contention overhead. The eliminated overhead is the result of backoff executed before a retransmission attempt is made when fragment transmission failure occurs within a fragment burst. This paper provides a mathematical analysis of BFFR energy efficiency and further assesses, by means of simulations, the energy efficiency, throughput and delay obtained when BFFR is used. The validity of the new scheme is evaluated in different scenarios namely, constant bit rate traffic, realistic bursty internet traffic, node mobility, rigid and elastic flows and their combinations at different traffic loads. We also evaluate and discuss the impact of BFFR on MAC fairness when the number of nodes is varied from 4 to 10. It is shown that BFFR has advantages over legacy MAC fragmentation scheme in all the scenarios.

## Background

Energy efficiency has impact on the global energy consumption and, as a result of energy generation process, the emission of carbon dioxide. In relation to information and communication technologies (ICT) two concepts have emerged namely, ICT for energy efficiency and energy efficient ICT. The latter is focused on the reduction of energy consumed by ICT systems which is reported, in several sources cited in Marsan and Meo (2011), to constitute 2–10 % of the global power and is expected to double over the next decade. About one third of this amount goes to networking (Marsan and Meo 2011). Our paper contributes to efforts towards achieving energy efficient ICT, specifically focusing on wireless networking.

IEEE 802.11 wireless local area networks (WLANs) have been widely deployed in public and private areas due to their low cost and improved throughput (He et al. 2008). More handheld devices, e.g. smartphones, are equipped with WLAN interfaces thus enabling users to access emerging mobile broadband Internet applications and services. Since such devices rely on a limited battery power, a substantial amount of research work has focused on reducing energy consumption and improving energy efficiency of WLANs devices at all layers of the TCP/IP protocol stack (Tsao and Huang 2011). We are interested in the medium access control (MAC) protocol and in particular the MAC layer fragmentation scheme. We have, therefore, designed and implemented an energy efficient fragmentation scheme for IEEE 802.11 based WLANs whose theoretical analysis, extensive and thorough performance assessment is the subject of this paper.

Fragmentation is a scheme designed to improve wireless data transmission whereby a large frame is split into a number of smaller ones (refereed to as *fragments*) which are independently transmitted and individually acknowledged. It has been studied, not only as an independent scheme in Filali (2005) and Tourrilhes (2001), but also in combination with rate adaptation in He et al. (2008), Kim et al. (2005) and Chang et al. (2007) for throughput improvement. Tourrilhes proposed a simple dynamic fragmentation scheme to deal with interference and thus improve throughput (Tourrilhes 2001). In this scheme the next fragment’s size is increased or decreased depending on whether the previous one was successfully or unsuccessfully transmitted respectively. The behavior of TCP in 802.11 based networks focusing on the effect of fragmentation and frame retransmissions done at MAC layer on the end-to-end TCP performance is examined in Filali (2005). Kim et al. (2005) proposed a dynamic fragmentation scheme whereby the transmission duration of all fragments, but the last one, is the same regardless the physical layer data rate. Different fragmentation sizes at different transmission rates are selected based on the channel condition. In this scheme, a new fragment is generated only when the rate for the next fragment transmission is decided. Throughput is the main performance metric.

While there have been a number of research papers pertaining to IEEE 802.11 MAC protocol energy consumption and efficiency, few have considered fragmentation from an energy efficiency perspective. Ramos et al. used the received signal strength to estimate bit error rate (BER) based on which the fragmentation threshold, transmit power or retry limit for the next transmission is decided Ramos et al. (2003). A survey of MAC layer energy efficient strategies for a WLAN station (STA) operating in continuous active mode (CAM) is reported in Tsao and Huang (2011). The strategies can be categorized into three. The first category consists of techniques which aim at conserving energy during contentions. The second category reduces energy consumption during transmission or retransmission of frames. Strategies in the last category aim at achieving energy efficiency by eliminating contentions, interframe space (IFS) and acknowledgement (ACK) messages. For each category there are a number of specific technologies proposed in the literature.

To reduce energy consumption of STAs operating in CAM the authors in Mafole et al. (2014a, b) selected the concept of eliminating contentions, IFSs and ACK. They proposed a fragment retransmission scheme that enhances the energy efficiency of WLANs by reducing contention overhead which STAs incur as a result of executing the backoff procedure whenever channel induced errors occur within a fragment burst. This scheme is named backoff-free fragment retransmission (BFFR). A performance evaluation of BFFR shows that it outperforms classical fragmentation (CF) scheme at different network sizes.

In Mafole et al. (2014a) the focus was on the superiority of BFFR over CF in both energy efficiency, and throughput at various network sizes. As an extension to Mafole et al. (2014a, b) showed that BFFR and CF have similar throughput and energy efficiency performance in free space, while in fading channels BFFR outperforms CF, at various network sizes. Moreover, Mafole et al. (2014b) further assessed the delay and fairness of BFFR in fading channels. In both papers, mathematical analysis to explain BFFR’s superiority over CF in energy efficiency is missing. Further, for performance evaluation in both papers, a constant bit rate (CBR) traffic was generated at the same intensity and was transported by UDP. CBR is a traffic generation model widely used in evaluating the performance of computer networks. In this paper, to further assess the validity of BFFR, we used a different traffic model which generates realistic Internet traffic. In previous papers, the performance of BFFR has been assessed when the STAs are fixed. However, the scheme is meant to be used in both fixed and mobile settings. It is, therefore, essential to assess BFFR by considering the impact of node mobility on its performance. In addition to this, since in a real network there exist both rigid and elastic flows, respectively associated with UDP and TCP flows, it is of interest to assess the performance of BFFR in such a scenario. We assessed BFFR when the traffic was offered in the form of rigid flows, elastic flows or a combination of both. The motivation for this paper is, thus, to complete the work in Mafole et al. (2014a, b) by providing a mathematical analysis of the scheme and evaluating the validity of BFFR in a number of scenarios not considered in the previous papers. We assess the energy efficiency, throughput and delay for different traffic types, offered load intensities and node mobility for both rigid and elastic flows.

This paper discloses interesting findings that were not observed in previous papers, namely the superiority of BFFR over CF when mobile STAs exchange realistic Internet traffic, at different offered load intensities, which is transported by UDP, TCP and their combination. The mathematical analysis syggests that the superiority is not limited to one PHY transmission rate but a all IEEE 802.11g PHY rates over a wide range of signal to noise ratio values. Our distinct contributions are therefore:

- A mathematical analysis which indicates that BFFR is superior to CF in energy efficiency in a rayleigh channel. The superiority is observerd in all IEEE 802.11g PHY transmission rates over a wide range of signal to noise ratios.
- The assessment of network performance when BFFR is used with two different traffic generators namely, the widely used CBR and a newly developed realistic internet traffic generator which is based on the Poisson Pareto burst process. The new traffic model captures key properties exhibited by real life IP traffic such as long range dependence and self similarity (Ammar et al. 2011). The model was validated against an autocorrelation function that reveals long range dependence property. Our simulation assessment, conducted at different offered load intensities, provides evidence that BFFR outperforms CF when subjected to real Internet traffic.
- We provide simulation based evidence that BFFR outperforms CF regardless whether the STAs are fixed or mobile, at average human walking speed, under different offered load intensities. We also show that BFFR has advantages over CF, in rigid and when rigid and elastic flows exist in a network, at different offered load intensities.

The rest of the paper is organized as follows: “MAC layer fragmentation scheme in IEEE 802.11 WLANs” section provides motivation for MAC layer fragmentation. We present a summary and a descriptive analysis of CF in “Classical fragmentation” section. The motivation for and a summary of BFFR is explained in “Backoff-free fragment retransmission” section and its mathematical analysis for energy efficiency is presented in “Analysis of BFFR energy efficiency” section. “Experiment setup and metrics definition” section describes the performance assessment setup while the obtained results and their discussion are presented in “Performance assessment results” section. We conclude our paper in “Conclusion” section.

## MAC layer fragmentation scheme in IEEE 802.11 WLANs

The rationale for including MAC layer retransmissions in WLANs as specified in IEEE 802.11-2007 (2007) is to avoid losing frames due to the occurrence of channel induced errors, collisions etc. The STA needs to retransmit the whole frame even if it contains only one bit error. In cases whereby the channel error rate is significantly high, to get the frame through would require a significant number of retransmissions and if the allowable maximum number of retries, namely, the retry limit *RL*, is reached the frame will eventually be dropped. To mitigate this, fragmentation was proposed whereby big frames are sent in small pieces (fragments) which are individually acknowledged or retransmitted. Doing this, in case of error the STA needs to retransmit only the error fragment which takes short time as compared to retransmitting the whole frame. If the medium is significantly noisy, a fragment has a higher probability to get through without errors because it can be fitted between error bursts (Tourrilhes 2001). By operating this way, the STA increases its chances of successful frame transmission in bad channel conditions.

### Classical fragmentation

The Distributed Coordination Function (DCF) is a compulsory and default MAC protocol in WLANs. DCF is a carrier sense multiple access with collision avoidance (CSMA/CA) scheme. Fragmentation is an optional DCF enhancement feature that can be enabled by specifying a fragmentation threshold (IEEE 802.11-2007 2007). When the size of a MAC service data unit (MSDU) arriving from the network layer is larger than that of the fragmentation threshold it will be split into fragments, MAC protocol data units (MPDU). The fragments belonging to the same frame are transmitted in a burst until all are sent or an *ACK* is not received. In either case the STA exits the burst and contends for the channel in accordance to CSMA/CA to send the next frame in the queue or retransmit a failed fragment respectively. In this paper, we refer to this as classical fragmentation (CF) scheme. Its operation is summarized in Algorithm 1. Despite its advantages, CF adds some overheads because it duplicates frame headers in every fragment and extra *ACK* for every successful fragment. Since an STA has to contend for channel whenever a fragment is to be retransmitted, further overheads are incurred due to backoffs.

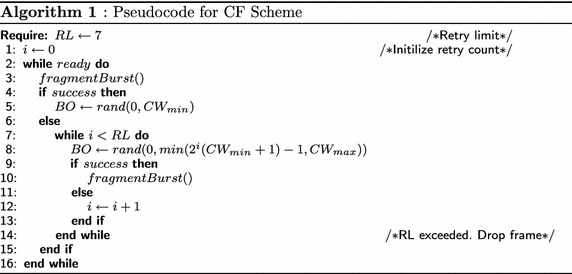



### Backoff-free fragment retransmission

During CF scheme, the first fragment and its corresponding *ACK* act as virtual channel reservation scheme by using the duration field in their MAC headers. The field defines the duration of the next transmission and its corresponding *ACK*. Neighbour STAs overhearing the transmission update their network allocation vectors (NAV) and defer from accessing the shared channel accordingly. For this reason collisions within a fragment burst are rare (Kim et al. 2005; Filali 2005). Thus transmission failures within the fragment burst are, at least in theory, mostly due to channel induced errors. During channel induced errors, the receiver silently discards the erroneous fragments (Vazifehdan et al. 2012). This means the receiving STA is *aware* of fragments received with errors (assuming the frame can be decoded) but *lets* the transmitting STA wait for $T_{EIFS}$. After the expiration of $T_{EIFS}$, prior to the failed fragment retransmission, the transmitting STA is required to contend for the medium in accordance to CSMA/CA.

1 $$T_{EIFS}= T_{SIFS} + T_{ack} + T_{DIFS}$$

2 $$T_{DIFS}= T_{SIFS} + 2\sigma$$

**Fig. 1:**
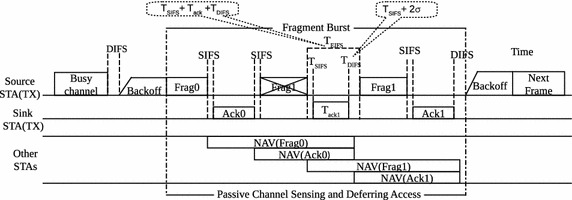
Retransmission of a failed fragment in BFFR

To prevent multiple STAs from owning the medium immediately after the completion of the preceding transmission, Eqs. 1 and 2 show relationships between time durations which must be adhered to by all STAs in a network. The parameters $T_{SIFS}$, $T_{DIFS}$, $T_{EIFS}$ and $\sigma$ denote time durations which are defined and fixed per physical layer (PHY) in DCF as stipulated in IEEE 802.11-2007 (2007). The symbol $\sigma$ stands for slot duration and $T_{ack}$ refers to *ACK* transmission duration. Since Eqs. 1 and 2 ensure that a message of length $T_{ack}$ can be sent from receiver to sender without collision before $T_{EIFS}$ expires, Mafole et al. (2014a) hypothesized that contention overhead can be reduced. A method to reduce the contention overhead was, therefore, proposed and implemented. Figure 1 shows a fragment burst within which a fragment is being retrasmitted according to BFFR whereby the receiver is tasked to notify the sender of an error fragment received so that it can be retransmitted within $T_{EIFS}$. Error notification is done over $T_{ack}$. In CF the $T_{EIFS}$ is wasted as the STAs involved in fragment transmission waits until the duration expires. This is followed by further time wastage as the STA enters backoff before attempting fragment retransmission.

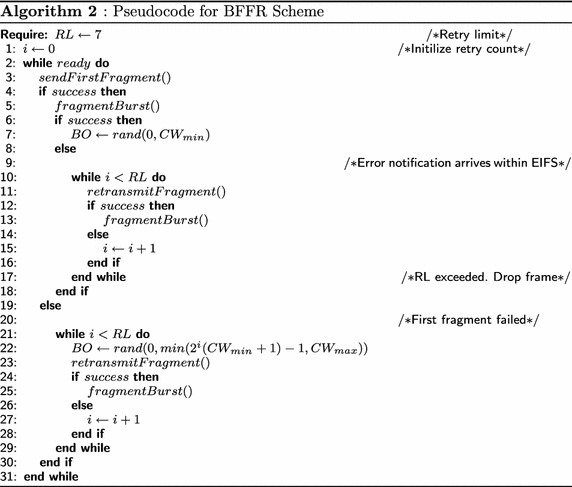



Upon successful notification the fragment is retransmitted, without channel contention, until *ACK* is received or a *RL* is reached. This is made possible by Eq. 2 which requires other STAs to wait for the medium to be idle for a $T_{DIFS}$, before they attempt to make a transmission, while the retransmission occurs a $T_{SIFS}$ after an STA has received the notification. This scheme is called backoff-free fragment retransmission (BFFR) fragmentation whose operation is shown in Algorithm 2. Its performance evaluation in different network sizes indicated that it outperforms CF (Mafole et al. 2014a, b).

## Analysis of BFFR energy efficiency

In this section we present the analysis of BFFR’s superiority over CF in energy efficiency. To carry out the analysis we use IEEE 802.11g standard parameters as shown in Table 1 where $N_{BPSC}$ and $N_{DBPS}$ refer to number of coded bits per subcarrier and number of data bits per OFDM symbol, respectively.

**Table 1:** PHY modes of IEEE 802.11g

Mode	Modulation	Code rate	Data rate (Mbps)	$N_{BPSC}$	$N_{DBPS}$
1	BPSK	1/2	6	1	24
2	BPSK	3/4	9	1	36
3	QPSK	1/2	12	2	48
4	QPSK	3/4	18	2	72
5	16-QAM	1/2	24	4	96
6	16-QAM	3/4	36	4	144
7	64-QAM	2/3	48	6	192
8	64-QAM	3/4	54	6	216

### Error performance over a Rayleigh channel

To begin the analysis, we derive the relationship between fragment error rate (*FER*) and signal to noise ratio (*SNR*) in a Rayleigh channel for each combination of modulation and coding scheme, *MCS*(*i*), as shown in Table 1 where $i\in \{1,2,3,4,5,6,7,8\}$. The *FER* depends on the performance of both, the demodulation and decoding (Schmidt 2012). The IEEE 802.11g OFDM PHY uses the Viterbi algorithm to decode convolutional codes. We derive the FER expression for the hard decision decoding *HDD* in a Rayleigh channel.

The Viterbi decoding algorithm provides an upper bound on the FER of an *l* sized fragment as given by Eq. 3 where $P_{e}$ is the union bound on the first-event error probability.

3 $$FER < 1 - (1-P_{e})^l$$

The demodulation bit error rate, *BER*, is adjusted to take into account error correction provided by convolutional coding (Schmidt 2012). With the Viterbi HDD, the union bound on the first event error probability $P_{e}$ is given by

4 $$P_{e} \le \sum \limits _{d=d_{free}}^{\infty }{\alpha _{d}*P_{2}(d)}$$

where $d_{free}$ is the free distance of the convolution code selected in *MCS*(*i*), $\alpha _{d}$ is the total number of error events of weight *d* and $P_{2}(d)$ is the probability that an incorrect path at distance *d* from the correct path being chosen by the Viterbi decoder. In principle, $P_{e}$ is evaluated by computing the sum of the pairwise error probability over all error events which correspond to a given transmitted sequence weighting each term by the number of information bit errors associated with that event. The weighted sum is then statistically averaged over all possible transmitted sequences, finally dividing it by the number of input bits per transmission (Simon and Alouini 2005). Tranfer function or numerical search methods can be used to otain $\alpha _{d}$ and $P_{2}(d)$ (Qiao and Choi 2001; Qiao et al. 2002). We follow the approach in Hepner et al. (2015) and Chen (2012) whereby it is approximated by summing up the first dominant terms. In Chen (2012) the first 10 terms are considered. $P_{2}(d)$ is given by

5 $$\begin{aligned} P_{2}(d) = {\left\{ \begin{array}{ll} \sum \limits _{z=\frac{(d+1)}{2}}^{d}{{d\atopwithdelims ()z}\rho ^{z}(1-\rho )^{d-z}}, & \quad \hbox{ if } d \hbox{ is odd}.\\ \frac{1}{2}{d\atopwithdelims ()d/2}\rho ^{(d/2)}(1-\rho )^{d/2} + \sum \limits _{z=\frac{d}{2}+1}^{d}{{d\atopwithdelims ()z}\rho ^{z}(1-\rho )^{d-z}}, &\quad \hbox{ if } d \hbox{ is even}. \end{array}\right. } \end{aligned}$$

where $\rho$ is the bit error probability for the selected *MCS*(*i*). A Rayleigh channel corresponds to a Nakagami-*m* fast fading channel with $m=1$. Following the analysis in Simon and Alouini (2005), the average *BER* of *N*-quadrature amplitude modulation (*N*-*qam*) of Nakagami-*m* fast fading channels with $m = 1$ is given by:

6 $$\begin{aligned} BER_{Nqam}&= {\scriptstyle 4\bigg (\frac{\sqrt{N}-1}{\sqrt{N}}\bigg )\bigg (\frac{1}{log_{2}N}\bigg )\sum \limits _{h=1}^{\sqrt{N}/2}\frac{1}{2}\Bigg (1-\mu _{h,Nqam}\sum \limits _{q=0}^{m-1}{2q\atopwithdelims ()q}\bigg [\frac{1-\mu _{h,Nqam}^2}{4}\bigg ]^q\Bigg )\Bigg |_{m=1}} \\&= {\scriptstyle 4\Big (\frac{\sqrt{N}-1}{\sqrt{N}}\Big )\Big (\frac{1}{log_{2}N}\Big )\sum \limits _{h=1}^{\sqrt{N}/2}\frac{1}{2}\Big (1-\mu _{h,Nqam}\Big )} \end{aligned}$$

making

7 $$\rho = {\scriptstyle 4\Big (\frac{\sqrt{N}-1}{\sqrt{N}}\Big )\Big (\frac{1}{log_{2}N}\Big )\sum \limits _{h=1}^{\sqrt{N}/2}\frac{1}{2}\Big (1-\mu _{h,Nqam}\Big )}$$

where:

8 $$\mu _{h,Nqam} = \sqrt{\frac{1.5(2h-1)^2\gamma BT_{b}k}{m(N-1)+1.5(2h-1)^2\gamma BT_{b}k}}$$

**Fig. 2:**
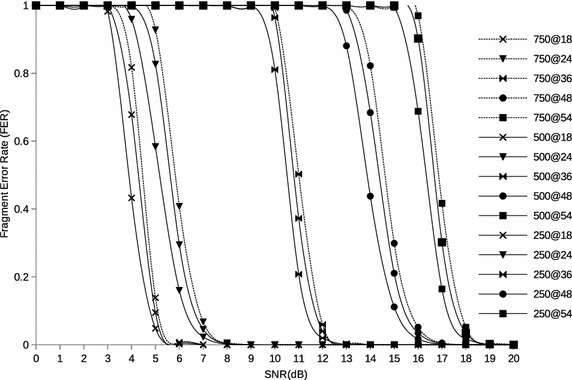
Fragment error rate in a Rayleigh channel

In Eq. 8, *k* is the number of bits per modulated symbol which is determined by the modulation scheme used in the transmission of a fragment. $T_{b}$ is the transmission duration per bit and *B* is the channel bandwidth, which is 20 MHz for 802.11g OFDM PHY. $\gamma$ is *SNR* given by:

9 $$\gamma = \frac{E_{b}}{N_{o}BT_{b}}$$

where $E_{b}$ is the energy per bit and $N_{o}$ is the noise density in *W*/*Hz*. With the average demodulation BER in Eq. 7, the FER can be calculated by using Eqs. 3, 4 and 5. Figure 2 indicates the FER for different fragment sizes over a range of signal to noise ratios as the framents are sent at different PHY transmission rates. We show results for *MCS*(*i*) where $i\in \{4,5,6,7,8\}$. It can be seen that, for a given PHY transmission rate, smaller fragments (250 Bytes) can withstand low values of SNR. Also higher PHY teansmission rates are prone to errors.

### Channel contention and backoff in DCF

The behavior of 802.11 DCF mechanism in error free channels has been analysed in several previous papers most of which are based on the seminal work by Bianchi (2000) and later extended and improved in Wu et al. (2002), Ni et al. (2005), Lee et al. (2006) and Tinnirello et al. (2010) to mention those relevant to this paper. We use main results from these works and their improvements, particularly the limited retry limit and the impact of channel induced errors. Channel induced errors have significant impact on energy consumption therefore it is neccessary to include them in our analysis.

We consider a WLAN with *n* STAs. When an STA experiences a transmission failure, either due to collision or channel induced errors, it enters backoff for a random number of slots, $T_{BO}(m)$, given by Eq. 10 where *m* is the *m*th retry, *RL* is the retry limit, ${CW_{min}}$ and ${CW_{max}}$ are minimum and maximum contention windows respectively.

10 $$T_{BO}(m)= \frac{1}{2}\sum \limits _{j=1}^{m-1}{\hbox{min}[2^{j-1}(CW_{min}+1)-1,CW_{max}]}, \quad 0< \hbox{ m }\le RL$$

We assume one of the *n* STAs attempts a transmission randomly. A collision occurs when at least one of the remaining $n-1$ STAs transmits. The probability of a collision is therefore:

11 $$p_{col,i} = 1 - (1-\tau _{i})^{n-1}$$

where $\tau _{i}$ stands for the probability an STA transmits in a general slot (Bianchi 2000; Wu et al. 2002; Ni et al. 2005; Lee et al. 2006; Tinnirello et al. 2010) and is given by Eq. 12.

12 $$\tau _{i} = \frac{2(1-p_{fail,i}^{m+1})(1-2p_{fail,i})}{T_{BO}(m)(1-(2p_{fail,i})^{m+1})(1-p_{fail,i})+(1-2p_{fail,i})(1-p_{fail,i}^{m+1})} \quad \hbox{ for } m\le RL$$

The term $p_{fail,i}$ stands for the probability of trasmission failure, due to either channel induced errors or collions, and is given by Eq. 13.

13 $$\begin{aligned} p_{fail,i}&= 1 - (1-p_{col,i})(1-p_{er,i}) \\&= 1 - (1-\tau _{i})^{n-1}(1-p_{er,i}) \end{aligned}$$

To calculate $p_{er,i}$, the probability of failure due to channel induced errors, we assume data and acknowledgment frames are corrupted independently of each other (Ni et al. 2005) leading to:

14 $$p_{er,i} = p_{er,i}^{data} + p_{er,i}^{ack} - p_{er,i}^{data}p_{er,i}^{ack}$$

where $p_{er,i}^{data}$ and $p_{er,i}^{ack}$ are obtained from Eq. 3. Since we are interested in the analysis of BFFR in a channel where fragment transmissions fail due to channel induced errors, we calculate probability at least one STA makes a transmission, the probability that at least one transmission made is successful and the probability of a successful transmission in an error prone channel by using Eqs. 15, 16 and 17, respectively, in a similar way to Lee et al. (2006).

15 $$p_{tr,i}= 1 - (1-\tau _{i})^{n}$$

16 $$p_{trs,i}= \frac{n\tau _{i}(1-\tau _{i})^{n-1}}{1-(1-\tau _{i})^{n}}$$

17 $$p_{trs*,i}= p_{trs,i}(1-p_{er,i})$$

Equations 12 and 13 form a set of non-linear equations which we solved numerically using *scilab* to obtain $\tau _{i}$ after which Eqs. 11, 15, 16 and 17 can be solved to obtain the respective probabilities.

### Frame transmission duration under fragmentation

To obtain the duration needed to transmit a frame under fragmentation we follow the analysis in Qiao and Choi (2001), Qiao et al. (2002) and Zhou and Kunisa (2010). The time duration for a data frame and an acknowledgement frame is given by Eqs. 18 and 19 respectively. $T_{data}(l,i)$ is the time duration of a data frame of length *l* sent by using *MCS*(*i*). The length *l* is comprised of the packet, MAC header and FCS field. The terms $T_{preamble}$, $T_{signal}$, $T_{sym}$ and $T_{sigExt}$, respectively, refer to preamble duration, signal duration, OFDM symbol duration and signal extension duration.

18 $$T_{data}(l,i)= T_{preamble} + T_{signal} + T_{sym}*\frac{(16 + 8*{l} + 6)}{N_{DBPS}(i)} + T_{sigExt}$$

19 $$T_{ack}(i)= T_{preamble} + T_{signal} + T_{sym}*\frac{134}{N_{DBPS}(i)} + T_{sigExt}$$

During fragmentation, the duration of an *l* bits fragment sent by using *MCS*(*i*), including the reception of its corresponding *ACK* message, is given by:

20 $$T_{frag}(l,i) = 2T_{SIFS} + T_{data}(l,i) + T_{ack} + 2\delta$$

where $\delta$ is propagation delay. If failure occurs, a fragment is retransmitted until either an ACK is received or a retry limit is reached. If retransmision is successful after $m-1$ retries, i.e at the *m*th attempt, then time to deliver the fragment becomes:

21 $$T_{fragRetry}(l,i,m) = T_{frag}(l,i) + T_{waste}(l,i,m)$$

where:

22 $$T_{waste}(l,i,m) = (m-1)({T_{data}(l,i) + T_{EIFS}} + 2\delta ) + \sigma (i)\sum \limits _{j=1}^{m-1}{T_{BO}(j)}$$

The expected duration of sending *l*-bit fragment under a retry limit, *RL*, such that $m\le RL$ is thus:

23 $$T_{fragRLSucc}(l,i,RL) = \sum \limits _{m=1}^{RL}{p_{succ}\big (m|l,i,RL\big )}T_{fragRetry}(l,i,m)$$

where $p_{succ}\big (m|l,i,RL)$, computed by Eq. 24, is the probability that a fragment is successfully delivered at the *m*th retry given that it will be delivered within *RL*.

24 $$p_{succ}(m|l,i,RL) = \frac{\big (1-p_{er,i}(l)\big )p_{er,i}^{m-1}(l)}{1-p_{er,i}^{RL}(l)}$$

For a wireless link with channel induces errors, there are cases whereby a fragment is eventually dropped after a retry limit has been reached. In such cases the corresponding time duration, all wasted in this case, is given by:

25 $$T_{fragDrop}(l,i,RL) = RL\big ({T_{data}(l,i) + T_{EIFS}+2\delta }\big ) + \sigma (i)\sum \limits _{j=2}^{RL}{T_{BO}(j)}$$

We assume a fragment burst has $N_{frags}$ = *L*/*l* fragments where *L* is the MSDU size and *l* is a fragment size. Considering the fact that any currupted fragment in a burst causes the STA to exit the burst, the duration to deliver *Nfrags* fragments is given by:

26 $$\begin{aligned} T_{N_{frags}}&= \sum \limits _{k=1}^{N_{frags}}{p_{succ}^{k-1}(1-p_{succ})\big ((k-1)T_{fragRLSucc}+T_{fragDrop}\big )} \\&\quad+\, p_{succ}^{N_{frags}}(N_{frags}T_{fragRLSucc} -T_{SIFS}) \end{aligned}$$

And finally, the total time duration to transmit all fragmnets of frame in a burst of $N_{frags}$ is given by:

27 $$T_{frame} = \sigma (i)T_{BO}(1) + T_{DIFS} + T_{N_{frags}}$$

### Energy efficiency under fragmentation in a Rayleigh channel

A WLAN network interface card,(WNI), can be in idle, transmit, receive and overhearing state. It, therefore, consumes energy which is a function of power spent in a given state and the duration over which the WNI remains in that state (Wu et al. 2011; Lee et al. 2006). To obtain the energy consumption in a given state we compute the product of power and the duration over which the power has been spent by NIC in that state. STAs contend for a time slot to send a fragment of a frame as described in “Channel contention and backoff in DCF” section. The slot could be idle or busy depending on the events in the wireless channel. We identify the events, compute their probabilities of occurence (using Eqs. 11, 13–17) and the energy consumed during each event.

#### Energy efficiency in classical fragment retransmission

In any slot possible events, from a transmitting STA point of view, include: finding an idle slot, making a successful transmission, experiencing a failed transmission due to channel induced errors, experiencing a failed transmision due to collisions, receiving a frame and overhearing a transmission. We compute the energy consumed by WNI during these events using Eqs. 28–33, respectively. In these equations $P_{idle}$, $P_{tx}$, $P_{rx}$ stand for power consumption of the WiFi radio in idle, transmission and receiving states respectively.

28 $$E_{idle}= \sigma P_{idle}\sum \limits _{j=1}^{RL}T_{BO}(j)(1-p_{col,i})(1-p_{er,i})(1-p_{tr,i})p_{fail,i}^{j-1}$$

29 $$\begin{aligned} E_{succ}&= \big (T_{data}(l,i)P_{tx} + T_{ack}(i)P_{rx} + (T_{SIFS}+T_{DIFS}+2\delta )P_{idle}\big ) \\&\quad *(1-p_{col,i})(1-p_{er,i}) \end{aligned}$$

30 $$\begin{aligned} E_{failErr}&= \sum \limits _{j=1}^{RL}j\big (T_{data}P_{tx}+(T_{EIFS}+2\delta +\sigma T_{BO}(j)\big )P_{idle})p_{err,i}^{data} \\&\quad+\, \sum \limits _{j=1}^{RL}j\big (T_{data}P_{tx}+(T_{ackTout}+T_{DIFS}+2\delta +\sigma T_{BO}(j)\big )P_{idle})p_{err,i}^{ack} \end{aligned}$$

31 $$E_{failCol}= \sum \limits _{j=1}^{RL}j\big (T_{data}P_{tx}+(T_{EIFS}+\delta +\sigma T_{BO}(j)\big )P_{idle})p_{col,i}$$

32 $$\begin{aligned} E_{recv}&= \big (T_{data}(l,i)P_{rx} + T_{ack}(i)P_{tx} + (T_{SIFS}+T_{DIFS}+2\delta )P_{idle}\big ) \\&\quad *(1-p_{col,i})(1-p_{er,i}) \end{aligned}$$

33 $$\begin{aligned} E_{overhear}&= \big ((T_{data}(l,i)+2T_{SIFS}+2\delta +T_{{ack}})p_{tr,i}p_{trs,i}p_{trs*,i} \\&\quad+\, (T_{data}(l,i)+T_{EIFS}+\delta )p_{tr}(1-p_{trs,i}) \\&\quad+\, (T_{data}(l,i)+T_{EIFS}+\delta )p_{tr}p_{er,i}\big ) \end{aligned}$$

To obtain energy efficiency we divide goodput by the sum of all the energy consumption components. For a frame of size *L* sent within a retry limit of *RL*, in fragments of size *l* at an arbitrary *MCS*(*i*), the goodput is given by Eq. 34

34 $$G(l,i,RL) = \frac{N_{frags}*l*p_{succ}^{N_{frags}}(l,i,RL)}{T_{frame}}$$

and energy efficiency is therefore given by Eq. 35.

35 $$E(l,i,RL) = \frac{G(l,i,RL)}{(E_{idle}+E_{succ}+ E_{failErr}+E_{failCol}+E_{recv}+E_{overhear})}$$

#### Energy efficiency in backoff-free fragment retransmission

The analyses in “Channel contention and backoff in DCF”, “Frame transmission duration under fragmentation” and “Energy efficiency in classical fragment retransmission” sections are in accordance to the classical fragmentation scheme as described in “Classical fragmentation” section. We now present the energy efficiency analysis of BFFR. To capture the behavior of BFFR as it is descibed in “Backoff-free fragment retransmission” section, Eqs. 22, 25 and 30 are modified and renamed as Eqs. 36, 37 and 38 respectively. It is worth noting that, in these equations, $T_{EIFS}$, is being used differently and STAs do not enter backoff when they experience fragment transmission failure within the fragment burst.

36 $$T_{wasteBffr}(l,i,m)= (m-1)(T_{data}(l,i) + 2T_{SIFS} + T_{ack} + 2\delta )$$

37 $$T_{fragDropBffr}(l,i,RL)= RL\big (T_{data}(l,i) + 2T_{SIFS} + T_{ack} +2\delta \big )$$

38 $$\begin{aligned} E_{failErrBffr}&= \sum \limits _{j=1}^{RL}j(T_{data}P_{tx}+(2T_{SIFS}+2\delta )P_{idle} + T_{ack}P_{rx})p_{err,i}^{data} \\&\quad+\, \sum \limits _{j=1}^{RL}j\big (T_{data}P_{tx}+(T_{ackTout}+T_{DIFS}+2\delta \big )P_{idle})p_{err,i}^{ack} \end{aligned}$$

All other equations, particulary Eqs. 26 and 35, derived from Eqs. 22, 25 and 30 are thus changed. We present the final equation for energy efficiency in BFFR as shown in Eq. 39.

39 $${E(l,i,RL)_{bffr} = \frac{G(l,i,RL)_{bffr}}{(E_{idle}+E_{succ}+ E_{failErrBffr}+E_{failCol}+E_{recv}+E_{overhear})}}$$

**Fig. 3:**
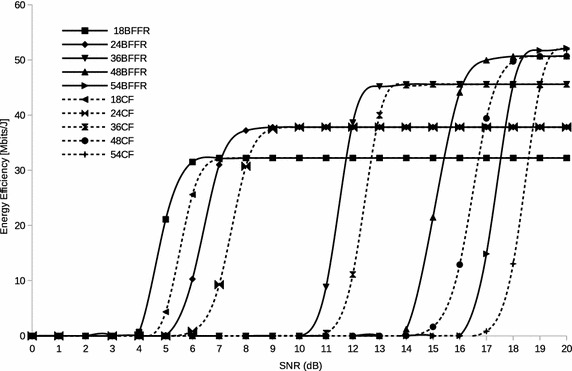
Energy efficiency of BFFR and CF in a Rayleigh channel

As it can be seen in Fig. 3, at a given PHY transmisssion rate, BFFR is superior to CF in a channel whose status is bad (represented by small values of SNR in the figure). At a given *MCS*(*i*), BFFR and CF attain the same value of maximum energy efficiency in a good channel status (represented by big values of SNR in the figure). In the next section we present results of simulation experiments we conducted to evaluate the validity of the proposed scheme in other scenarios different from those considered in Mafole et al. (2014a, b).

## Experiment setup and metrics definition

**Fig. 4:**
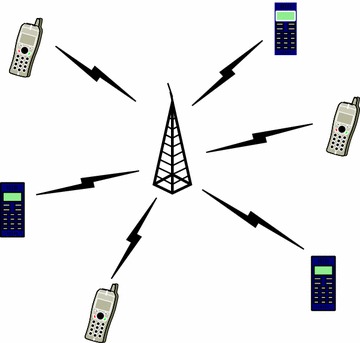
Network topology

To conduct performance assessment experiments we modified the MAC module of NS-3.14 to incorporate BFFR. We built up an infrastructure mode WLAN with a total of six STAs consisting of three transmitters and three receivers as shown in Fig. 4. In each run of the experiment the transmitters and receivers are randomly placed around the access point (AP) at a radius ranging from 10 to 60m. The STAs communicated over a fading channel modeled by chaining a Nakagami fading to a three log distance propagation loss model in order to obtain fading and path loss effects.

We adopted the energy framework presented in Wu et al. (2011) to simulate energy consumption of STA’s WNI. The framework consists of an energy source model for the supply of power to the STA and device energy model to imitate the energy consumption of the WNI. The device energy model captures the energy consumed by WNI during idle, busy channel sensing, sending and receiving states also when switching between them. Other simulation parameters are shown in Table 2. Simulation settings and configurations specific to a given scenario are provided in the respective subsections under “Performance assessment results” section.

**Table 2:** Simulation parameters

Parameter	Value
SIFS	10 $\upmu$s
DIFS	50 $\upmu$s
SlotTime	20 $\upmu$s
$CW_{min}$	15 Slots
Packet size	1500 Bytes
CCA threshold	−99 dBm
Transmission power	10 dBm
PHY transmission rate	54 Mbps
Energy detection threshold	−96 dBm

To validate the performance of BFFR against CF, as the baseline, we use three performance metrics namely, energy efficiency, throughput and delay. We measured throughput by counting the total number of useful data bits successfully received per unit time. Equation 40 shows the aggregate network throughput whereby *n* is the number of flows and $x_i$ is the throughput per flow. The quantification of energy efficiency was done by calculating the number of useful data bits in the *i*th flow, denoted by $X_i$, successfully received per one joule of energy consumed over the observation time. Equation 41 shows the network energy efficiency whereby energy consumed by a transmitter and receiver in *i*th flow is, respectively, given by $e_{t,i}$ and $e_{r,i}$. Delay is defined as the time duration that elapses from the moment a frame is queued for transmission to when it has been successfully received by the intended recipient. It includes queueing and MAC layer retransmission delays. The measurements are made per simulation run and an average over all the runs is reported.

40 $$\hbox {Throughput } = \sum \limits _{i=1}^n x_i \hbox { [Mbps] }$$

41 $$\hbox {Energy\,Efficiency } = \sum \limits _{i=1}^n\frac{X_i}{e_{t,i}+e_{r,i}} \hbox { [Mbits/J] }$$

## Performance assessment results

We present performance assessment results obtained in four scenarios namely, CBR traffic, realistic Internet traffic, node mobility, rigid and elastic flows at different offered load intensities. To further assess the validity of BFFR we use a different traffic model which generates realistic Internet traffic as explained in “Realistic internet traffic” section. BFFR has been assessed when the STAs are fixed. Since the scheme is meant to be used in both fixed and mobile settings, this paper completes the assessment by considering the impact of node mobility on the performance of BFFR. Moreover, since in a real network there exist both rigid and elastic flows it is of interest to assess the performance of BFFR in such a scenario. To implement BFFR, the behavior of DCF has been modified. Consequently it is of interest to investigate how the modification affects delay and fairness of medium access control. In “BFFR impact on delay and fairness” section we discuss the impact of BFFR on delay and present simulation results on DCF fairness when BFFR is being used and comapre it to when CF is being used. In the following subsections we detail each of these scenarios and discuss their respective performance assessment results.

### Constant bit rate traffic

In this section we assess the performance of BFFR as compared to CF at different CBR traffic intensities in a network consisting of 6 fixed STAs. The traffic intensity was varied from 128 kbps to 20 Mbps. CBR is often used to model real time applications such as voice and video. When transported over IP network these applications use UDP because reduced latency is of great importance to them. In our experiments, at a given offered load intensity, all CBR streams have the same data rate and they are transported over UDP.

**Fig. 5:**
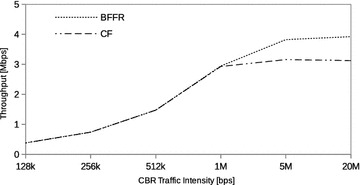
CBR traffic: throughput

**Fig. 6:**
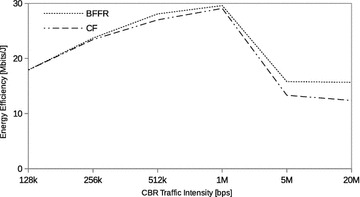
CBR traffic: energy efficiency

**Fig. 7:**
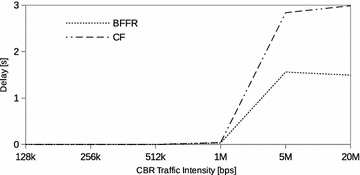
CBR traffic: delay

Figures 5, 6, and 7 respectively show the throughput, energy efficiency and delay performance of BFFR. It can be observed that BFFR maintains superior performance over CF when the offered load is at least 1Mbps for throughput and delay. As shown in Fig. 6, for energy efficiency, BFFR advantages can be observed when the offered load intensity is at least 256 kbps. From an end user’s point of view, this tentatively means, when it comes to multimedia services hungry handheld devices which rely on battery power, BFFR is a better candidate as compared to CF.

### Realistic internet traffic

The aim of this subsection is to validate BFFR by subjecting it to a traffic generator that matches key statistical properties of real life IP networks. We adopted such a traffic generator developed in Ammar et al. (2011). This generator captures important properties exhibited by real life traffic namely, long-range dependence and self-similarity. It uses a Poisson Pareto burst process (PPBP) to generate long-range dependent traffic. In this model bursts arrive according to a Poisson process with rate $\lambda _p$ whereby their length follows a Pareto distribution characterized by a Hurst parameter, *H* and a mean $T_{on}$. Each burst is modeled by a flow with constant bit-rate *r*. In aggregate, overlapping bursts form a long-range dependent traffic provided the burst lengths have infinite variance (Ammar et al. 2011). Traffic generator parameters used for validation experiments are shown in Table 3.

**Table 3:** PPBP traffic generator settings

Parameter	Value
$\lambda _p$	20
*H*	0.7
$T_{on}$	0.2 s
*r*	128, 512 kbps,..., 20 Mbps

**Fig. 8:**
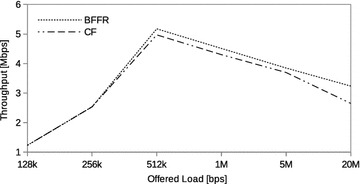
Realistic internet traffic: throughput

**Fig. 9:**
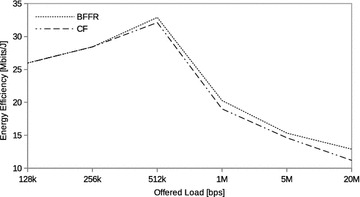
Realistic internet traffic: energy efficiency

**Fig. 10:**
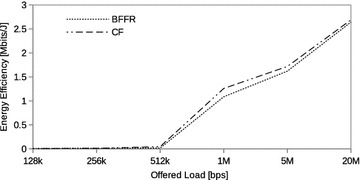
Realistic internet traffic: delay

Plots for throughput, energy efficiency and delay are shown in Figs. 8, 9 and 10 whereby BFFR has advantages over CF for offered loads as low as 256 kbps. The turning point is 512 kbps whereby both schemes have high energy efficiency, throughput and low delay. The observed general trend in performance metrics deterioration, for both BFFR and CF, is attributed to the increased collisions as the offered load intensity increases. This is because the level of collision-induced frame loss is dependent on both load and the number of stations (Leith and Malone 2010). In our experiments, the number of STAs was fixed.

Unlike the first, the second and subsequent fragments are protected from collisions by the Fragment−ACK handshake which also takes care of the hidden node problem. The way BFFR recovers from channel-error-induced frame loss, experienced by second and subsequent fragments, explains the observed improved performance in the metrics. CF has to contend for the channel to retransmit any fragment that is not acknowledged. BFFR, upon successful reception of error notification from the receiver, immediately retransmits the fragment without contending for the channel thus saving the time and energy that would otherwise be spent while in backoff. This results in higher throughput, energy efficiency and lower delay. The superiority of BFFR over CF when a realistic Internet traffic generator is used provides further evidence that, when it comes to multimedia services hungry handheld devices which rely on battery power, BFFR is a better candidate as compared to CF.

### Rigid and elastic flows

In order to validate a MAC protocol it is important to show that its performance for both elastic and rigid flows is acceptable (Barcelo et al. 2009). Elastic flows are associated with TCP. Good examples include file transfer, Web traffic and email. Rigid flows are associated with UDP and are exemplified by voice over IP (VoIP) applications. TCP and UDP flows have different requirements at the MAC layer (Barcelo et al. 2009; Choi et al. 2005) and thus the importance of validating a MAC protocol for both flow types. We configured a network that consisted of two rigid flows and one elastic flow. Offered load intensity was varied in a similar way, from 128 kbps to 20 Mbps. In our validation experiments we used TCP NewReno with segment size of 1448 bytes.

**Fig. 11:**
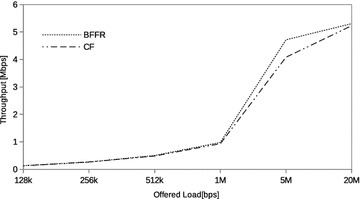
Mixed flows traffic: throughput

**Fig. 12:**
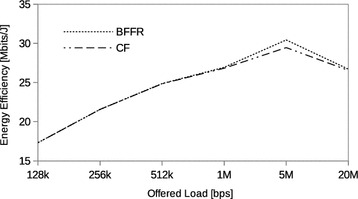
Mixed flows traffic: energy efficiency

**Fig. 13:**
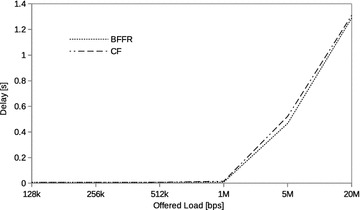
Mixed flows traffic: delay

Figures 11, 12 and 13 show the throughput, energy efficiency and delay when BFFR is used as compared to CF. It can be observed that, BFFR has advantages over CF when the offered load is between 1 and 20Mbps. This is tentatively due to the interaction between TCP congestion avoidance mechanism and BFFR as the offered load increases. We intend to investigate this further in our future work. On the other hand, this means BFFR was not only better than CF when it came to UDP traffic but also it did not adversely affect the performance of the network when both TCP and UDP traffic co-existed. In fact, BFFR was slightly better than CF in such scenarios. We, therefore, envisage that when BFFR is used, multimedia applications, file transfer, Web traffic and email can co-exist without adversely affecting network performance.

### STA mobility

So far, the STAs have been fixed. BFFR is designed to, energy efficiently, recover from channel induced errors without adversely affecting throughput and delay performance. Since node mobility has impacts on the link (leading to channel induced errors), protocols and application performance (Lenders et al. 2006) it is of interest to validate BFFR in this scenario as well. We used the 2D random walk mobility model available in NS3. The STAs move with a speed chosen randomly between 1.2 and 1.5 m/s. This is to simulate average walking speed of a human being. We selected the average walking speed of a human being so that it is in agreement with the values used in Nicolau and Jorge (2012). In our experiments, STAs mobility is bounded in a square of 60 × 60 m. Within this boundary, the mobile STA changes its current direction after every 5m moved in a random direction.

**Fig. 14:**
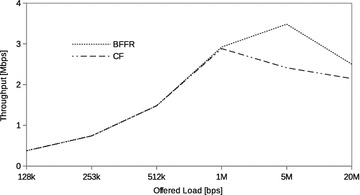
STA mobility: throughput

**Fig. 15:**
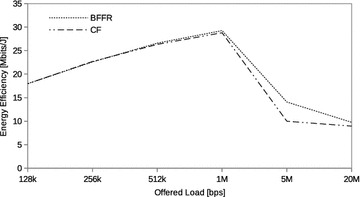
STA mobility: energy efficiency

**Fig. 16:**
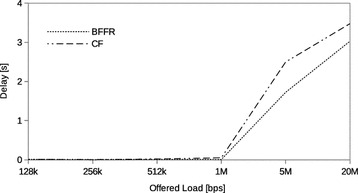
STA mobility: delay

The stability and superiority of BFFR over CF when STAs are in motion is shown in Figs. 14, 15 and 16 whereby BFFR maintains its performance gains in throughput, energy efficiency and delay respectively. Unlike other assessment scenarios, highest throughput and highest energy efficiency occur at different offered loads namely, 5Mbps and 1Mbps respectively. Comparing Figs. 5, 6 and Figs. 14, 15 we observe that the performance is better when nodes are fixed. This shows that node mobility has impact on the link and that BFFR is superior to CF in both scenarios.

To windup “Performance assessment results” section, we note that, in all the scenarios there exists an optimum offered load intensity at which highest energy efficiency is achieved. Since this did not always align with maximum throughput nor with minimum delay, our ongoing work is on the optimization of BFFR.

### BFFR impact on delay and fairness

**Fig. 17:**
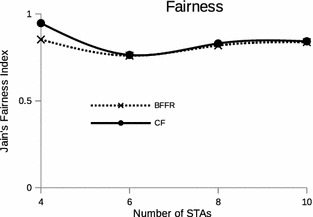
MAC fairness under BFFR scheme

A MAC protocol is fair if it provides channel access to individual STAs without giving preference to one STA over others when there is no explict differentiaon. One goal of a MAC protocol is thus to achieve good throughput while ensuring fair medium access (Bredel and Fidler 2009). Since we modified the behavior of DCF to implement BFFR, it is of interest to find out how fairness is affected. We conducted simulation expriments whereby the number of STAs was varied from 4 to 10 so that we could study fairness of medium access control as competition for the channel is increased. The number of STA was changed by adding a pair of STAs at a time (transmiter and receiver). Figure 17 shows Jain’s fairness index, a measure used to evaluate the degree of fairness of a MAC protocol. It can be seen in the figure, BFFR is less fair in a specific case of two transmissting STAs competing for the channel. However, in the general case of more that ten STAs BFFR’s fairness is the same as that of CF. This calls for optimization of BFFR parameters such as the retry limit value etc.

We have conducted extensive simulation experiments to study delay, retransmission ratio and energy efficiency of BFFR and results have been disseminated through Mafole et al. (2016). We observed that, as expected, different flows experience different delays as they go through different channel conditions in time and space. Moreover, as compared to CF the observed delay was lower or equal to that obtained when BFFR is being used in all the flows and at different fragmentation thresholds. This means the improvement in delay performance is across nodes.

## Conclusion

This paper has analysed the energy efficiency of BFFR as compared to CF over a wide range of SNR values and diffrerent transmission rates. We have provided a mathematical basis which shows that BFFR is superior to CF. Moreover, the paper has assessed the performance of BFFR in different scenarios namely, traffic types, STAs mobility and transport protocols. BFFR is a modification of MAC layer fragmentation scheme that, without contending for the channel, immediately retransmits a fragment lost due to channel induced errors. We have used CBR and a newly developed realistic Internet traffic generator to assess the performance of BFFR. In both cases, BFFR outperformed CF in all performance metrics considered. Moreover, when STAs are mobile BFFR is more stable against link quality variations due to node mobility and thus the observed better performance as compared to CF. Regarding the presence of mixed transport protocols in a network, UDP and TCP, BFFR has shown advantages over CF. We can therefore make a case that, BFFR is a better candidate than CF for multimedia applications when STAs are both fixed and mobile. It can as well be used in scenarios of mixed traffic consisting of multimedia applications, Web traffic, email and file transfer while and improve network performance currently achieved by CF.
